# Tissue-specific roles of *de novo* DNA methyltransferases

**DOI:** 10.1186/s13072-024-00566-2

**Published:** 2025-01-17

**Authors:** Dániel Márton Tóth, Flóra Szeri, Mária Ashaber, Muhyiddeen Muazu, Lóránt Székvölgyi, Tamás Arányi

**Affiliations:** 1 Department of Molecular Biology, Semmelweis University, Budapest, Hungary; 2 Institute of Molecular Life Sciences, HUN-REN Research Centre for Natural Sciences, Budapest, Hungary; 3 Department of Molecular and Nanopharmaceutics, Genome Architecture and Recombination Research Group, Faculty of Pharmacy, MTA-DE Momentum, University of Debrecen, Debrecen, Hungary

**Keywords:** DNA methylation, *De novo* methyltransferase, Dnmt3a, Dnmt3b, Knockout, Cre recombinase, LoxP, Tissue-specific, Development, Differentiation, Stem cells

## Abstract

DNA methylation, catalyzed by DNA methyltransferases (DNMT), plays pivotal role in regulating embryonic development, gene expression, adaption to environmental stress, and maintaining genome integrity. DNMT family consists of DNMT1, DNMT3A, DNMT3B, and the enzymatically inactive DNMT3L. DNMT3A and DNMT3B establish novel methylation patterns maintained by DNMT1 during replication. Genetic variants of *DNMT3A* and *DNMT3B* cause rare diseases such as Tatton-Brown-Rahman and ICF syndromes. Additionally, somatic mutations cause common conditions such as osteoarthritis, osteoporosis, clonal hematopoiesis of indeterminate potential (CHIP), hematologic malignancies, and cancer. While DNMTs have been extensively studied in vitro, in early development and in disease, their detailed physiologic roles remain less understood as in vivo investigations are hindered by the embryonic or perinatal lethality of the knockout mice. To circumvent this problem, tissue-specific *Dnmt3a* and *Dnmt3b* knockouts were engineered. This review explores their diverse molecular roles across various organs and cell types and characterizes the phenotype of the knockout mice. We provide a comprehensive collection of over forty tissue-specific knockout models generated by *cre* recombinase. We highlight the distinct functions of DNMT3A and DNMT3B in germ cells, early development, uterus, hematopoietic differentiation, musculoskeletal development, visceral organs, and nervous system. Our findings indicate that DNMT3A primarily regulates hematopoietic differentiation, while DNMT3B is crucial for cartilage homeostasis and ossification. We emphasize the context-dependent roles of DNMT3A and DNMT3B and demonstrate that they also complement DNMT1 maintenance methyltransferase activity. Overall, the expression patterns of DNMTs across tissues provide insights into potential therapeutic applications for treating neurologic diseases, cancer, and osteoporosis.

**Supplementary Information:**

The online version contains supplementary material available at 10.1186/s13072-024-00566-2.

## Introduction

### DNA methylation

The methylation of genomic DNA is the result of the addition of a methyl group on the C5 position of cytosine (5mC). This is the most extensively studied covalent chromatin modification, an epigenetic factor with multiple physiologic and pathologic roles. DNA methylation is crucial during development, ensures stable gene repression, maintains genomic integrity, establishes X chromosome inactivation, and regulates genomic imprinting [1].

In the mammalian genome, cytosines followed by guanines (CpG dinucleotides) serve as primary targets of DNA methyltransferase (DNMT) enzymes. However, non-CpG methylation also occurs in specific cell types, such as oocytes, pluripotent embryonic stem cells (ESCs), differentiating cells, and mature neurons [2–4]. CpG dinucleotides are unevenly distributed in the genome. The genome-wide CpG density is about ten times lower than expected. This results from the frequent 5mC to T transition, which is inefficiently repaired due to the natural presence of thymine in the DNA. The genomic density of CpG dinucleotides is almost equal to or even higher than expected in CpG islands (CGI). CGIs, often located in gene promoters, are frequently unmethylated whereas the vast majority of remaining CpGs are methylated, resulting in approximately 70% methylation of all CpGs in the human genome in somatic cells [5].

DNA methylation represents the most stable epigenetic mark. However, under physiologic, pathologic, and certain environmental conditions, CpG methylation levels can undergo rapid changes [6–8]. The most well-known example is embryonic development. Shortly after fertilization the paternal genome undergoes rapid and extensive demethylation, which is followed by a slower demethylation of the maternal genome [1, 5]. As a result, a significant global loss of genomic methylation occurs before implantation. However, there are species-specific differences, and the exact molecular mechanisms are still under investigation. Further, the role of loss of DNA methylation in the acquisition of pluripotency is still not clarified. This early demethylation is followed by a wave of remethylation upon implantation and the initiation of differentiation when totipotent cells become pluripotent. Interestingly, while the pluripotent cells of mesoderm lineage have a higher methylation level, cells of the visceral endoderm reach only a lower methylation level [9]. The neural lineage pluripont cells are less sensitive to the remethylation, but undergo significant methylation increase after birth [10, 11]. Lineage-specific methylation differences are particularly characteristic at enhancers. During embryogenesis primordial germ cells (PGC) undergo an even more important demethylation than the first wave during preimplantation. This demethylation is again followed by remethylation during complete maturation of the germ cells.

Demethylation can be either replication-dependent or independent [12]. Replication-independent demethylation is a complex, multistep oxidative process catalyzed by ten-eleven translocation (TET) proteins [13]. This active demethylation carried out by TET1, 2, or 3 dioxygenases is α-ketoglutarate, Fe^2+^, and ascorbate dependent. The TET enzymes oxidize 5-methylcytosine to 5-hydroxymethylcytosine then to formyl- and carboxylcytosine, ultimately leading to the replacement of methylated cytosine with unmethylated cytosine through base excision repair [13–15]. TET enzymes dynamically control gene expression and counterbalance the repressive effects of DNMTs, which is crucial to maintain cellular identity and transcriptional flexibility. Interestingly, the physiologic effect of TETs is both antagonistic and cooperative with that of DNMTs as this will be illustrated later in this review. Indeed, for a complete differentiation and maturation of various cell types (e.g. hematopoietic cells) both methylation and consecutive hydroxymethylation causing oscillatory cyclic DNA methylation changes particularly in pluripotent stem cells, is fundamental [16]. We have gained insights into these physiologic roles by studying the conserved TET (described elsewhere e.g. in [17, 18] and DNA methyltransferase (DNMTs) enzyme families. This review summarizes below the DNMTs and focuses specifically on two members of this family.

## DNA methyltransferases (DNMTs)

Following DNA replication, methylation is preserved on the original DNA template strand, while the newly synthesized strand remains unmethylated [19–22]. Without DNA methyltransferase activity, replication-dependent demethylation would occur. The DNMT1 maintenance methyltransferase accumulates at the hemimethylated double stranded DNA near the replication fork and re-establishes the original methylation pattern. In this process, UHRF1 plays a critical role by first binding to the hemimethylated CpG dinucleotides and then forming a complex with DNMT1. The cooperation of multiple epigenetic factors and UHRF1 ensures the accurate transmission of silenced chromatin structures through cell division [23].

In contrast to the other members of the DNMT family, DNMT2 stands apart by encoding an enzyme dedicated to methylating tRNA molecules [24].

The DNMT family is complemented by proteins primarily involved in *de novo* methylation. DNMT3A and DNMT3B establish DNA methylation profiles in differentiating stem cells, primordial germ cells during early embryogenesis, and to a lesser extent in differentiated cells later in life [25, 26]. These enzymes share similar domain organization (ADD, PWWP, and methyltransferase) with a very high sequence identity (∼85%) in the methyltransferase catalytic domain.

The expression of *Dnmt3a* and *Dnmt3b* starts already before implantation of the embryo [27]. These enzymes have a prominent role in repeat element silencing in ESC. The expression of the *de novo* DNA methyltransferases increases around implantation, and they carry out a very quick remethylation of the embryonic and extraembryonic lineages in postimplatation embryos. These enzymes are expressed in the pluripotent stem cells, which is maintained through adulthood. While in the different tissues their expression decreases with differentiation, the exact expression pattern is still unclear. Similarly to somatic cells *Dnmt3a* and *Dnmt3b* are expressed in PGCs where they are responsible for the remethylation of the cells through maturation.

**Fig. 1:**
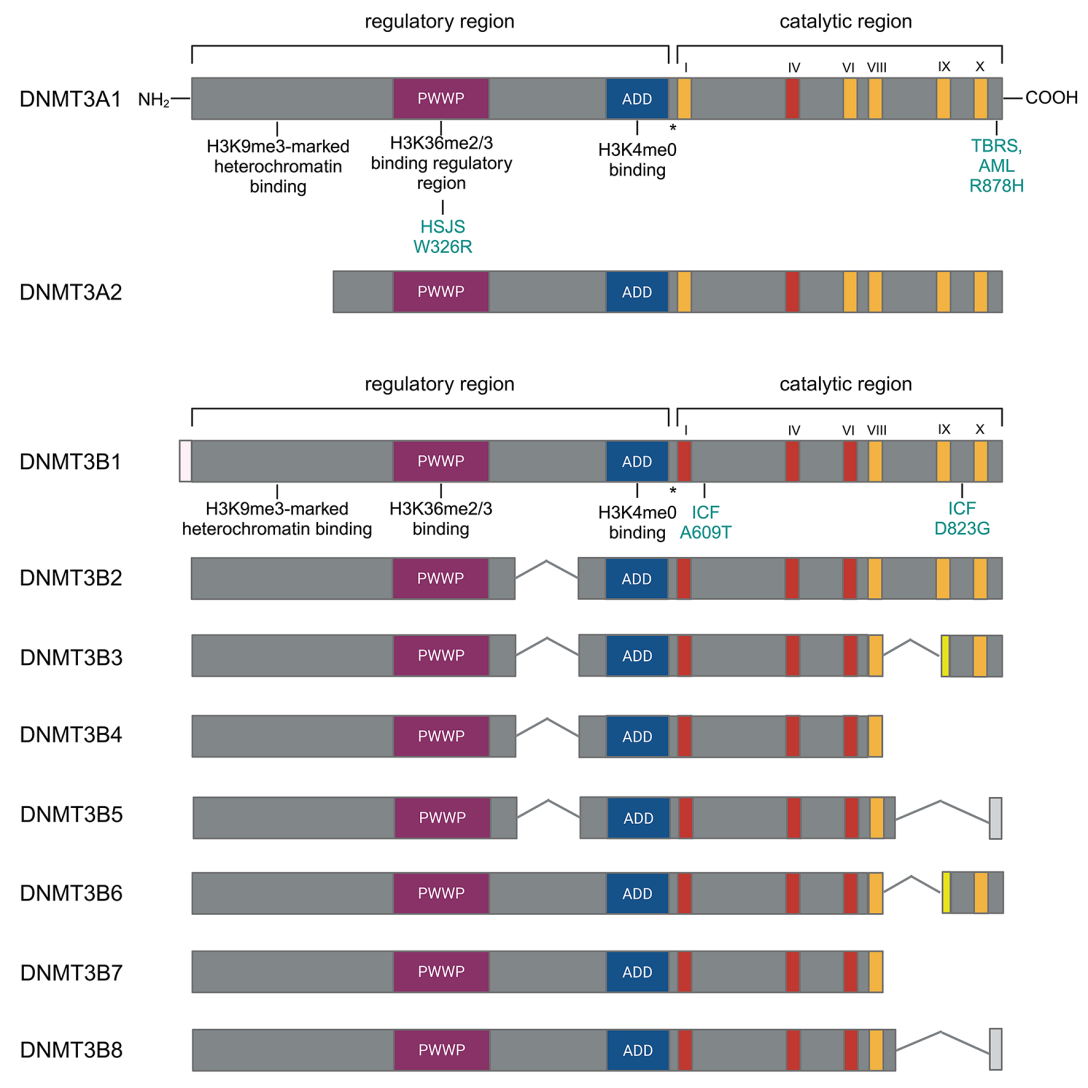
DNMT3A and DNMT3B isoforms in mouse. Conserved regions are colored, and their respective functions are indicated below. The conserved domain IV in the catalytic region of *Dnmt3a* is flanked by the loxP sites is indicated in red. HSJS stands for Heyn-Sproul-Jackson syndrome, associated with mutations in the DNMT3A PWWP domain. The W326R mutation is homologous to the human W330R disease-causing mutation and recapitulates the disease phenotype (see under “Medical Relevance” chapter). TBRS represents Tattoon-Brown Rahman Syndrome, AML indicates acute myeloid leukemia. Both can develop due to the dominant R382H mutation in human. The corresponding mouse mutation R878H is indicated. DNMT3B isoforms are shown on the panel below. The conserved domains I - VI in the catalytic region of Dnmt3b flanked by the loxP sites are indicated in red. A609T and D823G mutations cause similar phenotype than the human ICF syndrome (immunodeciency-centromeric instability-facial dismorphism). Homologous A603T and D817G mutations were reported in ICF patients

The expression of *Dnmt3a* is controlled by alternative promoters that produce two different isoforms: the full-length DNMT3A1 and the short variant DNMT3A2, which lacks an N-terminal domain [28] (Fig. 1). DNMT3A1 is expressed ubiquitously at low levels and localizes to heterochromatin, while DNMT3A2 is expressed at high levels in embryonic stem cells and is restricted to tissues undergoing *de novo* methylation such as the testis and the ovary, where it localizes in euchromatic regions [29]. Additionally, it has been shown that DNMT3A1, but not the short DNMT3A2 is essential for mouse postnatal development by binding to and regulating bivalent neurodevelopmental genes in the brain [30].

DNMT3B isoforms are shown on the panel below. The conserved domains I - VI in the catalytic region of *Dnmt3b* flanked by the loxP sites are indicated in red. A609T and D823G mutations cause similar phenotype than the human ICF syndrome (immunodeciency-centromeric instability-facial dismorphism). Homologous A603T and D817G mutations were reported in ICF patients.

In contrast to *Dnmt3a*, there are more than 30 splice variants of *Dnmt3b*, with only a few detected at the protein level and even fewer being catalytically active [31] (Fig. 1). *Dnmt3b1* and *Dnmt3b2* code for an intact C-terminal and enzymatically active catalytic domain. *Dnmt3b1* is usually regarded as the typical gene product of *Dnmt3b*. However, in male germ cells, *Dnmt3b2* was detected as the main transcript that can carry out *de novo* methylation [29]. The other six more abundant isoforms (Dnmt3b3-8) have deletions in the C-terminal catalytic domain, making them smaller and catalytically inactive proteins. However, their expression patterns are highly conserved between human and mouse, suggesting that these isoforms are biologically relevant. Indeed, they can act as accessory proteins that interact with catalytically active *de novo* methyltransferases and allosterically activate DNMT3B1 [32, 33].

Both DNMT3A and DNMT3B target genomic regions without methylation on either of the two strands. They methylate cytosines at CpG dinucleotides and non-CpG sites (CpH, mainly CpA), albeit with much lower efficacy for the latter [2–4]. The enzymes exhibit some, but fairly limited, sequence specificity regarding the neighboring nucleotides [34, 35]. Instead, histone modifications play a more important role in recruiting or inhibiting the binding of *de novo* methyltransferases to specific genomic regions.

H3K4me3, a hallmark of active promoters, and heterochromatin domains characterized by H3K27me3 or H2AK119 monoubiquitylation in general inhibit *de novo* methylatransferase activity. Conversely, H3K9me3 modifications (characteristic of pericentromeric heterochromatin), and H3K36me3 (present in actively transcribed gene bodies) actively recruit *de novo* DNMTs leading to heavy DNA methylation of these genomic regions. DNMT3A and DNMT3B recognize H3K36me2/3 through their PWWP motif (Pro-Trp-Trp-Pro) and confine transcription initiation to canonical transcription start sites (TSSs) by methylating intragenic promoters [36]. The *de novo* DNMT activity is increased by DNA methyltransferase 3-like (DNMT3L), a catalytically inactive but essential member of the DNMT family. DNMT3L acts as a bridging partner of *de novo* DNMT3s, particularly in germ cells between the target region and the enzyme [37–39]. DNMT3L selectively binds through its ADD (ATRX, DNMT3 and DNMT3L) domain to unmodified H3K4, while the binding is blocked by H3K4me3 [40]. In addition to these family members, DNMT3C completes the *de novo* methyltransferase subfamily in *Muridae* where it is specifically expressed in male germ cells and methylates transposons [41].

The critical physiologic roles of DNMT3A and DNMT3B in normal mammalian development were clear early on as evidenced by perinatal lethality in *Dnmt3a* knockout mice and embryonic lethality in *Dnmt3b* knockout mice. Disfunction of these enzymes has been linked to various human diseases, including acute myeloid leukemia (AML), ICF (immunodeficiency, centromere instability and facial anomalies) syndrome, and cancer (see under the Medical Aspects chapter). To elucidate the detailed physiologic roles of the *de novo* methyltransferases, tissue-specific knockout mouse models were engineered. These models have unveiled significant phenotypic traits providing valuable insights into the function of these enzymes, which will be comprehensively examined in the subsequent sections.

## Tissue-specific de novo DNMT knockout mouse models

*Dnmt1*, *Dnmt3a*, and *Dnmt3b*, are indispensable during development making challenging to explore their global and tissue-specific functions in vivo. Conditional knockout systems provide a targeted approach to overcome this limitation by allowing spatial and temporal control over gene deletion. This strategy allows one to unravel the involvement of DNMTs in lineage specification, differentiation, and maintenance of cellular identity.

The Cre/loxP recombination system is widely used to generate conditional knockouts (cKOs) in mammals [42]. In this method, Cre recombinase excises DNA segments flanked by loxP sites, with its expression directed by tissue-specific or inducible promoters for precise gene deletion. Alternative systems like Flp/FRT are employed in some contexts but are less prevalent [43]. Other methods like Zn finger nuclease (ZFN) [44] and TALEN (transcription activator-like effector nuclease)-mediated knockout [45] are also used sometimes for genome engineering. Recently, CRISPR/Cas9 has been adapted for conditional gene editing by enabling tissue-specific expression of Cas9 or guide RNAs, offering greater precision and flexibility [46]. However, this approach remains labor-intensive.

cKO systems face notable challenges. Leaky Cre expression can cause unintended recombination in off-target tissues or stages, resulting in mosaicism or unexpected phenotypes. Incomplete recombination may leave residual target gene expression, complicating phenotypic analysis. The efficiency and specificity of Cre recombinase depend heavily on the chosen promoter, introducing variability in gene deletion. For inducible systems like tamoxifen-activated Cre-ERT2, the timing of induction is critical, but inconsistencies in drug delivery or bioavailability can affect reproducibility.

Despite these limitations, cKO strategies have revolutionized the study of DNA methylation by circumventing embryonic lethality and enabling targeted gene disruption.

### Germ cells and early development

Numerous studies have been conducted to elucidate the role of DNMT3A and B during development. These investigations revealed that simultaneous deletion of *Dnmt3a* and *Dnmt3b* in embryonic stem (ES) cells leads to the loss of differentiation capacity [26]. Additionally, during the differentiation of single *Dnmt3a* or *Dnmt3b* knockout ES cells into mouse embryonic fibroblasts (MEF), significantly more DNMT3B- than DNMT3A-dependent cytosine methylation was identified [47]. Moreover, distinct gene sets specifically methylated by DNMT3A and DNMT3B were identified, with the DNMT3A-dependent gene set being notably enriched for developmental *Hox* genes.

**Fig. 2:**
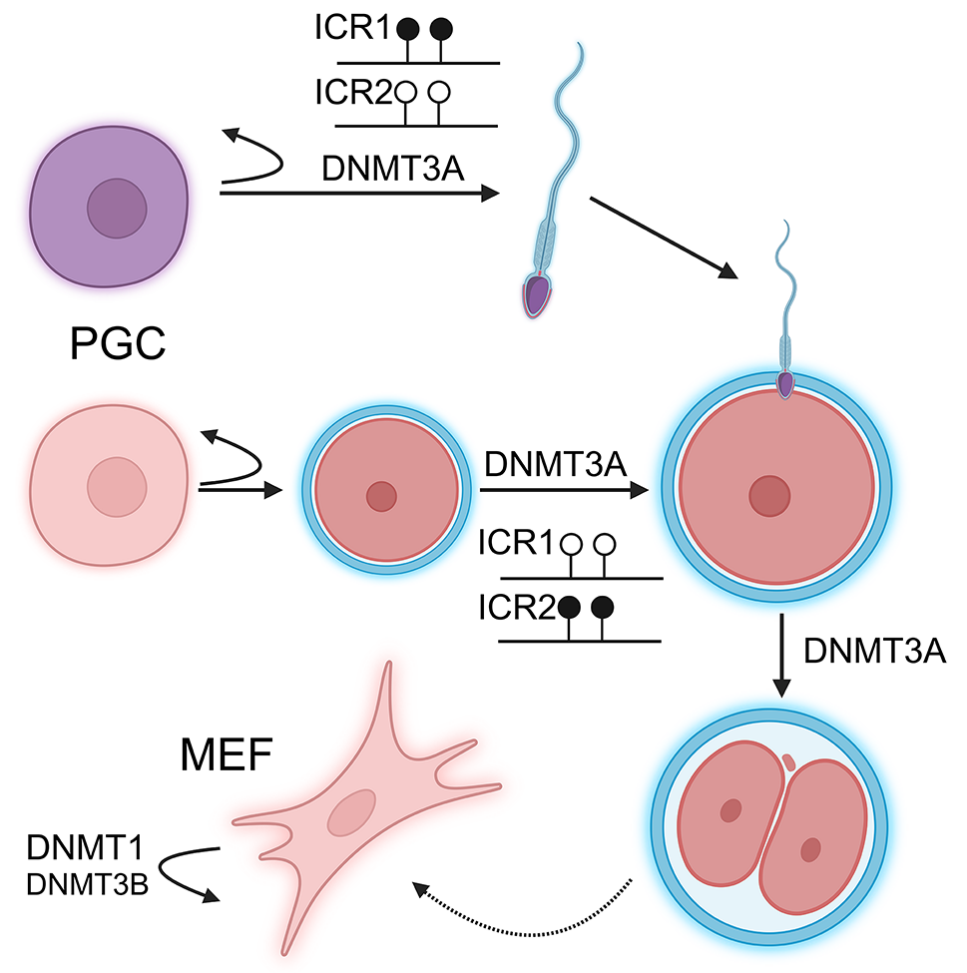
DNMT3A plays pivotal role in germ cell maturation and during preimplantation. *Dnmt3a* knockout primordial germ cells (PGC) are unable to differentiate to mature germ cells and can not establish parental-specific methylation patterns required for genomic imprinting. The PGCs maintain their ability to replicate and remain PGCs. DNMT3B plays minor role in complementing of the maintainance methylation activity of DNMT1 in mouse embryonic fibroblasts (MEF). ICRs are imprinting control regions. Lollipops represent CpG sites. Black CpGs are methylated, white CpGs are unmethylated

Although the requirement of *de novo* methyltransferases for differentiation is well established, the double knockout does not impede dedifferentiation and the formation of induced pluripotent stem cells (iPSC) from MEF [48]. Furthermore, the differentiation capacity of these cells could be restored by the reintroduction of the *de novo Dnmt*s. It has also been shown that in MEF cells DNMT3B exhibits some maintenance methyltransferase activity, which complements that of DNMT1 [49] (Fig. 2). In this knockout model, where *Dnmt3b* ablation was achieved by transfection of an adenovirus vector encoding *cre* recombinase, the methylation levels of retroviral and minor satellite repeats decreased in the absence of the *Dnmt3b* gene. The MEF cells exhibited either senescence or immortalization as well as chromosomal abnormalities, whereas knockout of *Dnmt3a* did not result in similar effects.

The *de novo* methyltransferases also play crucial roles in germ cells and early embryos [50] (Fig. 2). Particularly, spermatogonial stem cell specific knockout of *Dnmt3a* results in broad genomic demethylation highlighting the regulatory function of this enzyme [51] (Supplementary Table 1). In the absence of DNMT3A, the cells can maintain their stemness state but are unable to differentiate. Loss of DNMT3A in the oocyte, causes decreased methylation and impaired accumulation of 5-hydroxymethyl cytosines in the maternal genome after fertilization [7]. Moreover, similar effect of the maternal DNMT3A was observed on the paternal genome in the zygote and 2-cell embryo [7, 52].

Genomic imprinting, characterized by parent-of-origin specific expression of a few dozens of genes, is regulated by various epigenetic mechanisms, including DNA methylation. A hallmark of these genes is a regulatory region called the imprinting control region (ICR), which is methylated on the silenced allele and devoid of methylation on the transcribed allele (Fig. 2). Not surprisingly, the DNMTs play a role in this process. The initial studies using *TNAP-cre* to inactivate *Dnmt3a* and *Dnmt3b* in the germ cells demonstrated the necessity of DNMT3A for the methylation of both the maternal and paternal imprinted loci, while DNMT3B has no role in this process [53]. Subsequent studies confirmed that DNMT3B has no role in the methylation of ICR. Additionally, it has been shown, that after establishing the methylation pattern in the ICR, DNMT3A is not required for the maintenance of this methylation mark [54, 55]. However, during reprogramming of the pluripotent stem cells, DNMT3A is recruited to remethylate some ICRs [56].

### Uterus and placenta

**Fig. 3:**
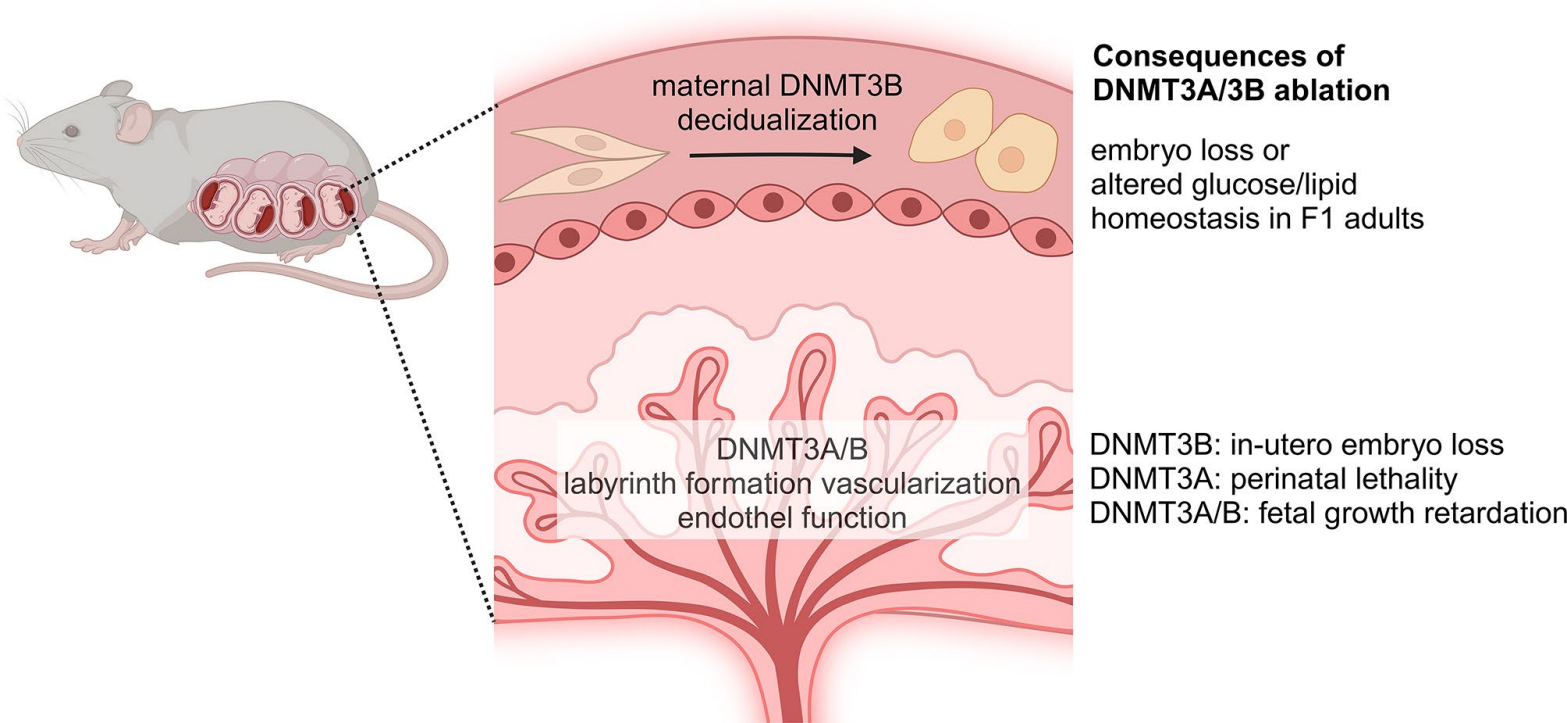
DNMT3A and DNMT3B are involved in fetal development. DNMT3B is required for the decidualization of the endometrium and the absence of the gene from progesterone-sensitive cells disturbs this process and leads to embryonic loss. Both DNMT3A and DNMT3B are required for labyrinth formation. The maintenance of *Dnmt3b* expression in trophoblast cells is crucial for embryonic development and survival

While DNMT3A was initially considered to play only minor role in the uterus [57], its ablation from placental vascular endothelium led to decreased vascularization, resulting in placental insufficiency and fetal growth retardation [58] (Supplementary Table 1). Similarly, depletion of *Dnmt3b* in progesterone receptor-positive uterine stromal and epithelial cells by *Pgr-cre* did not cause any major alteration at first glance [59]. However, upon more detailed analysis, it became evident that uterine knockout mice experienced decidualization of the endometrium, leading to the loss of approximately half of the implanted embryos (Fig. 3). Although this was accompanied by alteration in the uterus-specific gene expression pattern, no global DNA methylation changes were observed in the tissue. However, data on a handful of genes indicated an inverse correlation between RNA expression and DNA methylation changes. The physiologic relevance of the endometrial *Dnmt3b* expression was further underlined when the F1 generation was studied, as the offsprings of knockout dams were characterized by lipid and glucose homeostatic change and hepatic histologic alterations [60].

The study of the placenta, a complex organ composed of both maternal and fetal tissues, has recently shed light on the role of DNMT3B in the extraembryonic tissues. As mentioned earlier, whole body *Dnmt3b* ko is lethal *in utero*. This is consistent with the more significant decrease of methylation observed in full body *Dnmt3b* knockout compared to that of *Dnmt3a*. During embryogenesis, there is a substantial loss of methylation particularly in the extraembryonic tissues rather than the epiblast, in double *de novo methyltransferase* knockout (Fig. 3). Interestingly, in *Sox2-cre* mediated epiblast-specific *Dnmt3b* knockout embryos, where the expression of the gene in trophoblasts is maintained [61, 62], the survival rate is nearly normal. However, these mice die shortly after birth [62]. Collectively, these findings strongly suggest that the *in utero* lethality of *Dnmt3b* knockout is likely due to placental failure.

### Hematopoietic differentiation

**Fig. 4:**
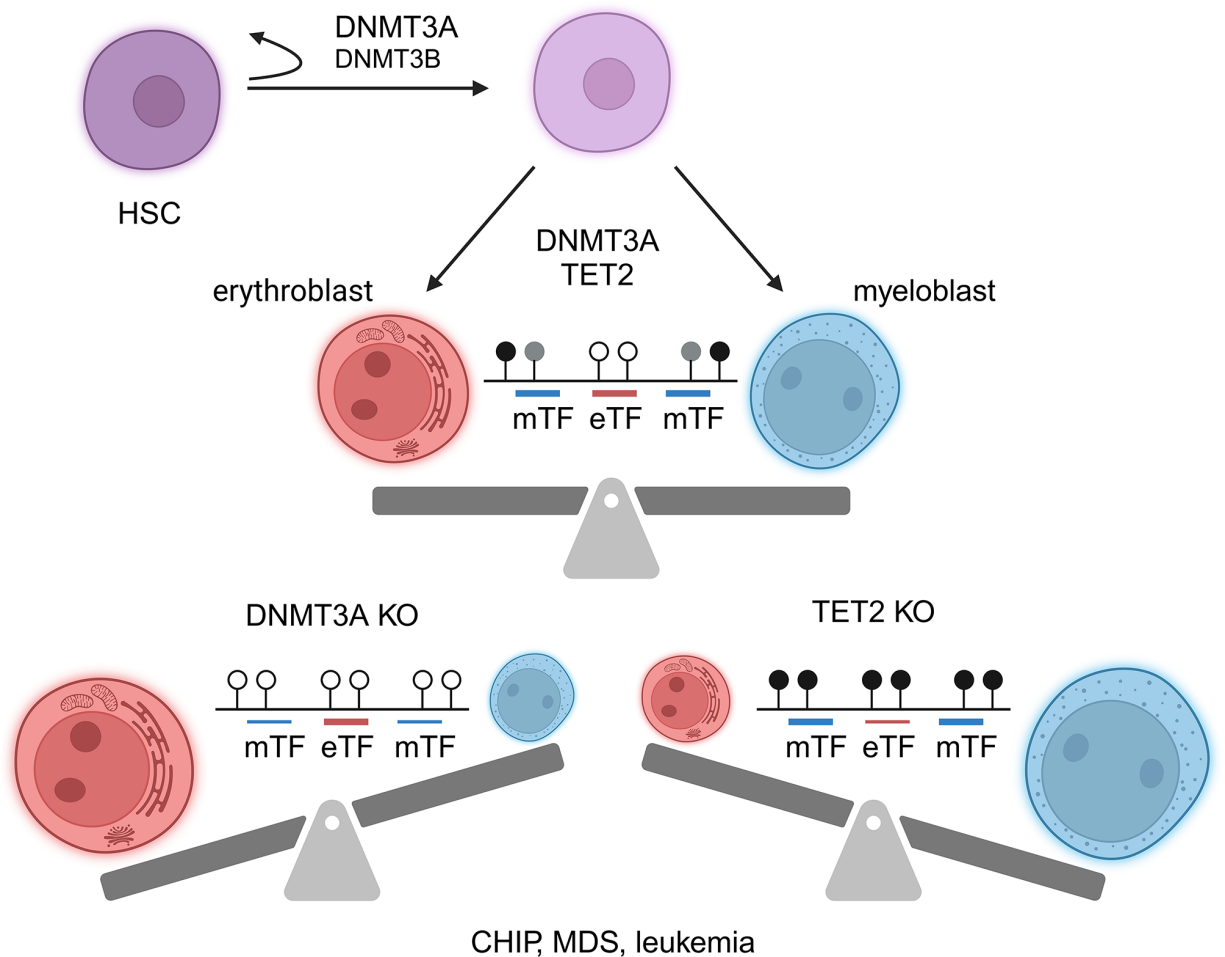
Hematopoietic stem cells (HSC) maintain their stemness or undergo differentiation after replication. Differentiation is primarily regulated by DNMT3A. DNMT3B has a complementary role in this process. Hematopoietic differentiation also requires TET2 demethylase activity. The methylome is characterized by kb long canyons with very low methylation levels enriched by methylation sensitive transcription factor (TF) binding sites involved in erythroid differentiation. The edges of the canyons are enriched in 5hmC (grey lollipops), regulating myeloid differentiation. TFs binding these regions are less sensitive to DNA methylation. *Dnmt3a* knockout mice have skewed erythroid, *Tet2* knockout mice have skewed myeloid differentiation. CHIP: clonal hematopoiesis of indeterminate potential; MDS: myelodysplastic syndrome

The role of *de novo* methyltransferases has also been traced through terminal differentiation with a particular focus on hematopoietic cells. Initially, studies showed that hematopoietic stem cells (HSC) lacking *Dnmt3a* [63, 64] and/or *Dnmt3b*, obtained by the transplantation of *Mx1-cre* mediated knockout HSC transplantation, experienced a loss of differentiation potential and showed enhanced self-renewal capacity [65] (Fig. 4). These investigations revealed a synergistic role between the two *de novo* methyltransferases, with DNMT3A playing a crucial role in hematopoiesis while DNMT3B primarily complements its function [66]. Unexpectedly, in the absence of either or both genes, distinct genomic regions experienced DNA hypermethylation. Additionally, more recent studies have demonstrated a methylation-independent regulatory role of DNMT3A in splicing in both ESC and HSC [67].

Numerous studies have explored the role of the *de novo* methyltransferases in the differentiation of various subsets of HSC employing different *cre* lines [68–75] (Supplementary Table 1). However, the real function of DNA methylation in hematopoiesis was only elucidated when the roles of DNMT3A and TET2 were compared in single or double knockout HSCs [76–78] (Fig. 4). In the absence of DNMT3A, the balance of erythroid/myeloid differentiation was skewed toward increased erythroid differentiation [77]. Conversely, the lack of TET2 led to increased myeloid differentiation. These studies also identified large (several kb long) unmethylated genomic regions, termed “canyons”, characterized by edges with high 5-hydroxymethyl cytosine content [76]. The center and edges of canyons are enriched in several transcription factor binding sites and include genes involved in hematopoiesis. Mutations of *Dnmt3a* in patients with hematologic malignancies were associated with gene expression alterations of canyon genes. Finally, it was found that a few transcription factors responsible for erythroid differentiation (Tal1, Gata1, and Klf1) comprise more CpG dinucleotides in their binding sites and are, therefore, more sensitive to DNA methylation than myeloid transcription factors [77]. Consequently, these recognition elements are more methylated in *Tet2* knockout cells and hypomethylated in *Dnmt3a* knockout cells, resulting in altered balance between unmethylated, methylated, and hydroxymethylated states compared to wild-type cells. This implies a regulated cooperation and competition between DNMT3A and TET2 [76, 78].

### Musculoskeletal system

**Fig. 5:**
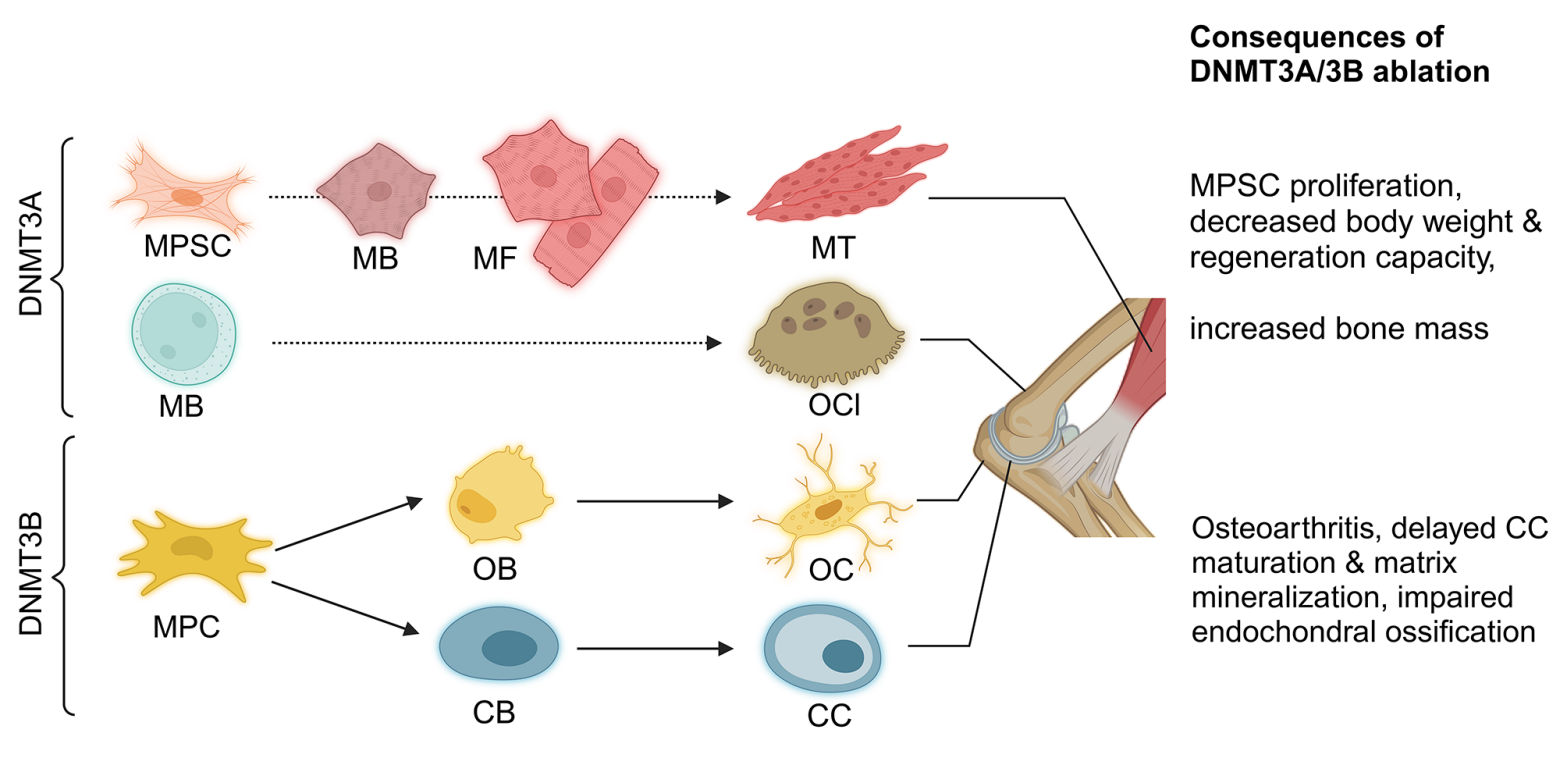
*De novo* methyltransferases play primordial role in ossification. Mesenchymal progenitor cells (MPC) differentiate to chondroblasts (CB) then chondrocytes (CC) and osteoblasts (OB) then osteocytes (OC). DNMT3B is a key regulator of this process. In the absence of Dnmt3b delayed CC maturation, osteoarthritis, impaired fracture healing is observed. Osteoclasts (OCl), responsible for bone resorption and maintaining homeostasis of ossification are differentiating from MPC (macrophages) themselves originating from myeloblasts (MB). In the absence of *Dnmt3a* increased bone mass was reported. Skeletal muscle development also requires DNMT3A. In knockouts decreased body weight was reported. MPSC: myogenic progenitor stem cells; MB: myoblasts; MF: myofibrillul; MT: myotube

After deciphering the role of the *de novo* methyltransferases during HSC differentiation and in various subsets of hematopoietic cells, their importance in other tissues was progressively unveiled (Fig. 5). It has been shown that unlike in hematopoietic differentiation, the loss of *Dnmt3a* has only minor effect in ossification, whereas DNMT3B plays a crucial role in this process [79]. The absence of *Dnmt3b* from chondrocytes induces osteoarthritis [79, 80] (Supplementary Table 1). Moreover, *Dnmt3b* deficiency in the embryonic chondrocyte lineage delayed chondrocyte maturation and matrix mineralization [81]. The *Dnmt3b* knockout also leads to other alterations, mainly due to the absence of the enzyme from the mesenchymal progenitor cells [82–84]. Impaired endochondral ossification, reduced fracture repair and decreased mechanical strength of the newly formed bone were also observed. The molecular mechanism is not yet fully understood but is likely based on increased Notch signaling, which is normally suppressed by DNMT3B. On the other hand, in the osteoclast precursor cells, originating from macrophages, the main *de novo* methyltransferase is DNMT3A [85]. Knocking out *Dnmt3a* in the osteoclast lineage by *Rank-cre* transgene results in osteoclast precursor cells failing to differentiate into mature osteoclasts, and the mice exhibit increased bone mass due to insufficient bone resorption. Surprisingly, under certain conditions some DNMT3A mutant can cause increased osteoclastogenesis and thus osteoporosis (see under the medical aspects).

Finally, the role of DNMT3A in the musculoskeletal system was also investigated in muscle-specific knockout mice [86]. Though minor genome-wide methylation loss and gene expression alterations were identified, no major effect of the gene knockout was reported. However, another model of tissue-specific knockout highlighted the function of *Dnmt3a* in skeletal muscle as well [87]. Muscle precursor satellite cell-specific *Dnmt3a* knockout mice by Pax3-cre transgene display decreased body weight, muscle mass, and impaired muscle regenerative capacity. The molecular mechanism behind could be that DNMT3A induces proliferation by inhibiting the expression of the cyclin-dependent kinase inhibitor (p57kip2), a cell-cycle regulator, in satellite cells. The increased expression of p57kip2 in the *Dnmt3a* knockout cells resulted in the inhibition of proliferation and eventually led to lower muscle mass [87].

### Visceral organs

The roles of the *Dnmt3a* and *Dnmt3b* were investigated during organogenesis and in response to environmental stimuli. Minor effects of the enzymes were detected across various germ layers: ectoderm-related tissues such as the lens [88] and epidermis [89]; mesoderm-related tissues including cardiomyocytes [90], kidney [91], and uterus (see above); and endoderm-related tissues such as the liver [92] (Supplementary Table 1). However, a more detailed analysis revealed their significant function in specific visceral tissues and cell-types.

For instance, depletion of *Dnmt3b* in cardiomyocytes resulted in altered mRNA splicing and the accumulation of alternatively spliced transcript variants of the sarcomeric *Myh7* gene, leading to compromised systolic function [93]. In vitro studies have demonstrated that DNMT3B and DNMT3L suppress the expression of cardiomyocyte marker genes in ESCs. Additionally, it has been observed that in the absence of *Pten*, a key regulator of cardiac differentiation, *Dnmt3b* expression increases concomitantly with CpG and non-CpG methylation, a characteristic effect of the *de novo* methyltransferases. This inhibits IGF2 expression, a downstream regulator of cardiomyocyte differentiation [94]. The double knockout of *Dnmt3b* and *Dnmt3l* by Crispr/Cas9 rescued the phenotype of the *Pten* knockout ESC cells, resulting in an increased number of cardiomyocytes in the culture under differentiation conditions. These findings were further confirmed in in vivo experiments.

In another knockout scenario, the significance of DNMT3B’s role was uncovered only upon subjecting the model to challenges. In the brown fat and skeletal muscle progenitor-specific *Dnmt3b* ablated mice (obtained by a *Myf5-cre* transgene) no phenotype was observed until the age of 4 months [95]. However, female mice without functional *Dnmt3b* were susceptible to obesity when fed a high fat diet (HFD), as they gained significantly more weight than their control littermates. These *Dnmt3b*-deficient females also developed insulin resistance, despite no increase in food consumption. Notably, only minor DNA methylation and metabolic gene expression changes were detected. Surprisingly, males showed resistance to HFD, which did not have any effect on the skeletal muscles either. It is important to highlight that the choice of transgenes used to drive the ablation of *Dnmt3b* is crucial. The same research group, shortly after these results, published another study using the *Ucp1-cre* transgene to eliminate *Dnmt3b* in mature brown adipocytes. In this study, females exhibited a lean phenotype, and contrasting effects on the expression of the majority of the same few genes investigated in the previous study were documented [96]. Interestingly, in both studies males were resistant to HFD challenge. This suggests that DNMT3B might have inverse epigenetic effects on gene expression and phenotype at different stages of differentiation, or that the different subset of affected cells may have antagonistic influences on the phenotype.

Other studies have shown that *Dnmt3a* also contributes to adipogenesis. In *Prx1-cre* mice, where *Dnmt3a* was deleted in adipocyte progenitor cells, increased progenitor cell number and larger fat deposition were observed in the aging males, especially in the subcutaneous regions [97].

The analysis of liver-specific knockout of *Dnmt3b* revealed only minor phenotypic effects [92]. Specifically, subtle genome-wide DNA methylation loss, accompanied by an altered expression of the oxidative phosphorylation genes were observed in adult knockout mice. However, the role of DNMT3B became evident when various challenges were applied. In primary hepatocytes from the knockout animals, thioacetamide (TAA) treatment reduced mitochondrial total oxygen consumption and increased the level of reactive oxygen species (ROS). The same study demonstrated that DNMT3B plays a protective role against hepatic inflammation, fibrosis, and carcinogenesis in the TAA-induced liver fibrosis model.

Similarly to other organs, an apparent lack of phenotype was reported in the intestinal epithelium of *Villin-creERT2* transgene-driven conditional tissue-specific *Dnmt3b* knockout mice [98]. The importance of the gene was revealed when studied together with *Dnmt1*. The single *Dnmt1* knockout was not lethal; nevertheless, it caused body weight decrease and DNA methylation loss, but the mice mostly recovered after two weeks. However, in the double knockout of *Dnmt3b* and *Dnmt1*, the phenotype was much more severe leading to 60% lethality, with some mice recovering due to some cells escaping *Dnmt3b* ablation. This suggests that DNMT3B has an important complementary maintenance methyltransferase activity. Surprisingly, the role of DNMT3B in DNA methylation was not analyzed in detail in this study, but it had no effect on LINE-1 loci and *H19*. Altogether, these data suggest that as discussed earlier for MEF cells and as we will see later as well, DNMT3B and DNMT1 have some overlapping, probably maintenance function. In the *Villin-creERT2* model *Dnmt3a* doesn’t seem to play any important role, as neither the single nor the double ablation with *Dnmt1* had any effect on the phenotype of the mice [98]. However, in another very similar model (*Villin-cre*), DNMT3A was found to be involved in the maintenance of colon epithelial integrity [99]. In these mice, the epithelial cells have shortened apical junctional complexes, and the colon has increased permeability, which might explain why these mice are more susceptible to colitis.

The roles of the *de novo Dnmt*s in the kidney were studied in two similar models, both investigating a double knockout *Dnmt3a* and *Dnmt3b* [91]. The ablation was performed either with a *Six2-cre* transgene expressed in nephron progenitor cells or with the *Ksp-cre* transgene expressed in both the developing and adult kidney. While the mice did not display any visible phenotype they exhibited genome-wide DNA methylation alterations, predominantly hypomethylation. These hypomethylated regions were preferentially located in enhancers, most of which were fetal-specific. Their hypomethylation was accompanied by gene expression alterations as well, including the increased expression of developmental genes. Interestingly, a significant portion of these hypomethylated regions overlapped with enhancers conserved in humans and showing fetal-type methylation pattern in diabetic nephropathy. This suggests that the *de novo methyltransferases* play a role in preserving the correct methylation pattern of these regions in humans to prevent the development of diseases. This underscores a potential specific role of DNMT3B, which also functions as a complementary maintenance methyltransferase.

The role of DNMT3A was also investigated in the endocrine pancreas. Mature pancreatic beta cells are characterized by glucose-stimulated insulin secretion (GSIS). To acquire this phenotype, the cells undergo a metabolic shift postnatally [100]. It has been shown that in beta cells, the only *de novo* methyltransferase expressed is *Dnmt3a* during the first 3 weeks of life. To explore the role of DNMT3A in this metabolic transition, cell-type specific ablation of the gene was performed *RIP-cre* (rat insuline promoter) or the beta-cell specific *PDX-cre ert2* [100]. Beta cells lacking *Dnmt3a* exhibited methylation alterations of key glycolytic genes, remained immature, and were unable to develop GSIS.

### Nervous system

While only minimal effect of *Dnmt3a* or *Dnmt3b* ablation in glial cells have been reported so far [101, 102] (Supplementary Table 1), several studies have highlighted the significant role of the *de novo* methyltransferases in neurons. Inducible deletion of *Dnmt3a* and *Dnmt3b* in adult hippocampal neuronal stem and progenitor cells (NSPCs), responsible for generating new neurons throughout life, indicated that *de novo* DNA methyltransferases are involved in the morphological and functional maturation of new neurons including dendritic outgrowth [103]. Furthermore, environmental enrichment, known to induce DNA methylation changes, resulted in fewer activated neurons in the NSPC knockout animals compared to the wild-type. In contrast, a higher percentage of activated interneurons were detected in the knockout animals. These alterations led to impaired learning and memory in behavioral tests in the *Nestin-cre ert2* mice. These findings underscore the role of *de novo* methyltransferases under stress conditions. Moreover, the crucial role of *Dnmt3a* during brain development is further emphasized as targeted deletion of *Dnmt3a* in the brain leads to reduced motor neuron numbers, accumulation of fragmented endplates in neuromuscular junctions, and in premature death with motor defects [104, 105].

However, *Dnmt3a* ablation from mid-gestation exclusively in excitatory neurons of the neocortex and the hippocampus [106] resulted in milder phenotype with altered behavior including impairments of working memory, and social interest, as well as inefficient synaptic maturation, and plasticity. Alongside these behavioral changes, the *Nex-cre* model exhibited other epigenetic changes at the molecular level. Notably, unmethylated regions were invaded by H3K27me3 signal and polycomb repression.

The significance of methylation in postmitotic neurons was first demonstrated by forebrain-specific double knockout of *Dnmt1* and *Dnmt3a*, revealing deficits of learning, memory, and synaptic plasticity [107]. Similarly, double deletion of *Dnmt1* and *Dnmt3a* by *AAV2/8-cre* in primary culture of hippocampal neurons reduced excitatory synapse formation, suppressed synaptic transmission and decreased neuronal activity [108]. However, DNMT1 and DNMT3A enzymes cannot functionally compensate for each other in postnatal neurons as evidenced by adult forebrain-specific single knockout mice. The lack of *Dnmt3a* led to impaired learning, memory, and synaptic plasticity, meanwhile *Dnmt3b* expression remained unaltered [109]. Nonetheless, DNMT3B also contributes to memory formation as its absence in dorsal hippocampal neurons leads to a deficit in object-place recognition memory as evidenced by *AAV-syn-cre* mediated knockout [110].

Furthermore, another intriguing observation regarding *de novo* methylation events in the brain is that conditional knockout of *Dnmt3a* in the preoptic area of newborn female mice results in subsequent male sexual behavior. This observation suggests that feminization is an active and ongoing repression of masculinization in the brain [111]. In addition, the role of *Dnmt3a* in the central nervous system was also shown in the regulation of energy and metabolic homeostasis. This was revealed by the deletion of the gene in different hypothalamic neurons. Its absence in the agouti-related protein (AgRP) expressing neurons leads to increased adiposity attributable to a reduced tendency for voluntary exercise [112], while its deficit in other hypothalamic neurons (Sim1 neurons within the paraventricular nucleus) manifests in obesity, hyperphagia, and glucose intolerance [113].

In conclusion, these studies highlight the crucial role of *de novo* methyltransferases, *Dnmt3a* and *Dnmt3b*, in embryogenesis, stem cell differentiation, and cellular responses to environmental stress. These enzymes exhibit partially overlapping but distinct cell-type specific physiologic regulatory function, which are being progressively elucidated. Additionally, both genes have been associated with various human pathologies. The final section of this review summarizes our current understanding of these diseases resulting from germ line or somatic mutations of *Dnmt3a* or *Dnmt3b*.

## Medical relevance

The diseases associated with mutations of the *de novo* methyltransferases can be categorized into two groups: hereditary syndromes and disorders resulting from somatic mutations or expression alterations. Studies in mice have demonstrated that whole-body knockout of either *Dnmt3a* or *Dnmt3b* is not compatible with life. Consequently, it is not surprising that the three documented syndromes of familial origin are very rare. Although these syndromes are characterized by partially preserved enzyme activity, the mutations result in symptoms that impact multiple organs.

The Tatton-Brown – Rahman syndrome [114, 115] is an overgrowth syndrome characterized by increased height and head circumference, often accompanied by moderate intellectual disability or autism spectrum disorder (ASD). This autosomal dominant condition is caused by *de novo* loss-of-function mutations of the *DNMT3A* gene (Fig. 1). Recent research suggests that the neurodevelopmental phenotype might be partially attributed to reduced neuronal non-CpG (CpA) methylation mediated by DNMT3A. Methylated CpA sites are recognized by MeCP2, and the absence of this epigenetic mark can lead to Rett-syndrome variants, similar to the classic Rett-syndrome caused by mutations of MeCP2 [116, 117].

The Heyn – Sproul – Jackson syndrome, unlike the Tatton-Brown – Rahman syndrome, results from gain-of-function mutations of the *DNMT3A* gene [118]. Mutations affecting the PWWP domain disrupt its interaction with the H3K36me2/me3 regions, causing methylation to spread to H3K27me3-decorated regions (Fig. 1). Individuals with this syndrome present microcephalic dwarfism accompanied by intellectual disability. Recent discoveries also link germline gain-of-function mutations of the DNMT3A PWWP domain to head and neck paragangliomas or papillary thyroid carcinoma [119–121]. However, the reason why some patients develop tumors instead of dwarfism remains unclear.

Hypomorphic recessive loss-of-function mutations of *Dnmt3b* lead to the development of immunodeficiency – centromeric instability – facial (ICF) dysmorphism syndrome [122] (Fig. 1). Individuals with this syndrome experience recurrent respiratory infections, mental retardation, and exhibit chromosomal abnormalities, typically fusion of chromosomes 1, 9, and 16 in lymphocytes. It should be noted that not all patients have *DNMT3B* mutations and even among those who do, the observed DNA methylation loss is generally mild.

Some polymorphisms in the regulatory region of *Dnmt3b* have been associated with autoimmune diseases, particularly Graves’ disease and Hashimoto thyroiditis [123]. However, the molecular pathomechanism behind this association remains unclear. While strong correlations have been observed between DNA methylation alterations and a wide range of chronic inflammatory and neurodegenerative diseases, the pathologic role of DNMT3A and/or DNMT3B in these condition has not been conclusively demonstrated.

It has also been shown that DNMT3B is crucial for maintaining pulmonary artery physiology. In patients with pulmonary hypertension, a condition with life-threatening implications, *DNMT3B* expression is increased in vascular smooth muscle cells. Intriguingly, studies using a rat model suggest that this increased expression may serve as a compensatory feedback mechanism. Indeed, *Dnmt3b* knockout rats showed facilitated development of pulmonary hypertension, a phenotype that could be prevented by overexpression of the gene [124].

Diseases related to somatic mutations or altered gene expression of DNMT3A or DNMT3B often impact bones, chondrocytes, and joints. In osteoarthritis there is an upregulation of *Dnmt3a* and *Dnmt1* expression. However, the significant decrease of *Dnmt3b* expression is more important in the disease pathogenesis as it impacts the TCA cycle and mitochondrial respiration [79, 125] (Fig. 5). This disruption of the homeostasis of articular chondrocytes contributes significantly to osteoarthritis development. The pathogenic role of DNMT3B was confirmed in a chondrocyte-specific knockout mouse model, which also developed osteoarthritis [79].

Osteoporosis and other related diseases characterized by increased bone resorption due to bone homeostasis alteration, is often observed in patients with clonal hematopoiesis of indeterminate potential (CHIP) associated with increased osteoclastogenesis [126] (Figs. 1 and 4, and 5). Dominant-negative mutations of *Dnmt3a* at the R882 hotspot are common in these patients. Mouse models of hematopoietic-specific *Dnmt3a* knockout [127] have also developed osteoporosis, providing additional evidence of DNMT3A’s involvement in the pathogenesis of the disease.

CHIP has also been linked to cardiovascular diseases [128, 129]. For instance, patients with CHIP often exhibit increased inflammation due to *Dnmt3a* mutations in monocytes and macrophages, leading to elevated level of inflammatory cytokines implicated in atherosclerosis, aortic stenosis, and heart failure. Similarly, the role of DNMT3A in mast cell-mediated allergic diseases, such as asthma, has also been proposed, though definitive evidence is still lacking [130].

The final large group of pathologies affected by the *de novo* methyltransferases includes hematopoietic malignancies [131], and solid tumors. DNMT3A have similar effects as a tumor suppressor in hematopoietic cells [132]; therefore, in 20–30% of various hematologic disorders *Dnmt3a* mutations seems to have a causal role in their development. In addition to CHIP, which slightly increases the risk of leukemia, myelodysplastic syndrome (MDS) [133, 134], acute myeloid leukemia (AML) [135, 136], acute lymphoid leukemia (ALL, T-cell ALL) [71, 135], and chronic lymphoid leukemia (CLL) occur frequently [137]. Various DNMT3A mutations can cause these diseases but the most frequent one is the dominant negative arginine to histidine mutation (R882H) (Fig. 1).

It has also been shown that the effect of the two *de novo* DNA methyltransferases is synergistic [66]. DNMT3B also has similar effects to a tumor suppressor in hematopoietic cells. Indeed, it has been shown that in mixed lineage leukemia (MLL-AF9), the *Dnmt3b* gene is downregulated and its deletion in mice causes a similar leukemia phenotype [66]. The role of DNMT3B was further highlighted in the development of T-cell lymphoma [138] in a mouse model recapitulating the human disease. In this model the loss of *Dnmt3b* in T-cells led to the progressive loss of DNA methylation at specific genomic regions (e.g., *Ment* gene), indicating the involvement of DNMT3B in maintenance methylation. This is in line with the secondary function of the enzyme described earlier.

Finally, somatic mutations or expression alterations of the *de novo* methyltransferases are also observed in various solid tumors. Interestingly, while the genome-wide methylation alterations in most tumor types have been extensively studied, the mechanistic roles of the *de novo* methyltransferases have been documented in relatively few reports. Furthermore, these enzymes are considered tumor suppressors in certain tissues and oncogenes in others, while DNMT3A and DNMT3B have occasionally opposite effects.

According to the NCI GDC Data portal (https://portal.gdc.cancer.gov [139] somatic mutations of *Dnmt3a* and *Dnmt3b* are present in approximately 3–5% of the tumors occurring across a wide array of tissues such as the skin, lung, GI tract, uterus, breast, prostate, and pancreas. The incidence of *Dnmt3a* mutations is generally somewhat higher than that of *Dnmt3b*. However, in most solid tumors, significant *DNMT3A* and/or *DNMT3B* expression alterations are associated with the disease.

The tumor suppressor effect of DNMT3A was demonstrated in a mouse squamous-cell carcinoma model, which became even more aggressive in the concomitant absence of *Dnmt3b* [89]. It has also long been known that the deletion of *DNMT3A* promotes lung cancer growth and progression [140]. In contrast, increased *DNMT3B* expression has been reported in colorectal cancer [141], while the deletion of *Dnmt3a* inhibits colorectal cancer development [142]. Functional DNMT3B however, is only necessary for macroadenoma formation in the intestines [143]. In both endometrial cancer [144] and hepatocellular carcinoma [92] an increased expression of *DNMT3B* was reported. Other studies reported altered *DNMT3A* and/or *DNMT3B* expression in ovarian [145], prostate [146–148], stomach [149, 150], and breast [151, 152] cancers, among others.

## Future directions

Future research will need to explore different directions. The studies compiled here utilized tissue-specific knockout models applying the cre-lox system. They showed the effect of the complete ablation of DNMT3A or DNMT3B either in the entire body or in various tissues. However, with the advent of the CRISPR base-editing technology, it is now possible to introduce point mutations and other sequence variations, as demonstrated in case of the residue R882, the mutational hotspot of DNMT3A linked to hematologic malignancies. Exploration of the mutational landscape of DNMT3A or DNMT3B could provide a more intimate understanding of the exact role of *de novo* methyltransferases. This approach may also shed light on the functions of their different domains and would even allow the refined discrimination between distinct roles related or not to the methyltransferase activity such as reported in [71].

Similarly, further mechanistic studies should be conducted to better understand the functional roles of the numerous splice variants of DNMT3A and particularly DNMT3B. A multitude of DNMT3B isoforms have been reported, and studies utilizing isoform-specific knockout mice would further our understanding of their physiologic and potential pathologic roles. Recent research has highlighted the significance of this field by demonstrating that postnatally knocking-out specific DNMT3A splice variants in mice lead to lethality, while the ablation of other splice variants results in no apparent phenotypic alteration [30].

Another aspect of future studies should focus on the role of DNMTs in the acquisition of pluripotent state. Why is it necessary for the early embryos to undergo global demethylation? Why do they undergo global remethylation?

And finally, the role of DNMT3A and 3B should be further dissected in the development of diseases. Are there other hereditary syndromes related to mutations of these genes? Are the alterations of the DNA methylation in common diseases and cancer causes or consequences of the developing clinical phenotypes?

## Electronic supplementary material

Below is the link to the electronic supplementary material.

Supplementary Material 1

## Data Availability

No datasets were generated or analysed during the current study.
