# Supplementary material for: Tissue-specific roles of de novo DNA methyltransferases
Source: Epigenetics Chromatin. 2025 Jan 17;18:5. doi: 10.1186/s13072-024-00566-2 (PMC11740433; doi:10.1186/s13072-024-00566-2)
Supplement: Supplementary file 1 — Supplementary Material 1 [file 13072_2024_566_MOESM1_ESM.pdf]

Supplementary Table 1. Cre constructs used for conditional knockout of Dnmt3a and Dnmt3b.

Transgene	Specificity	Comment	Reference
Prdm1-cre	primordial germ cell	*Blimp1-cre*<br>Commercially available	(51)
Zp3-cre	oocyte	Commercially available	(7, 52)
TNAP-cre	Embryonic primordial germ cell	*Alpl* gene<br>Commercially available	(53)
Pgr-cre	Uterus (stromal and epithelial cell)	Commercially available	(57, 59)
Cdh5-cre	endothel	Commercially available	https://doi.org/10.1101/2022.07.28.501807 (58)
Sox2-cre	epiblast	Commercially available	(62)
Mx1-cre	Interferon α/β inducible	Commercially available	(63-65)
Esr-cre	Estrogen-inducible	Estrogen Receptor-β<br>Commercially available	(66)
Mb1-cre	B lymphocytes	CD79a<br>Commercially available	(69)
CD19-cre	B Lymphoid cells	Commercially available	(70, 71)
CD4-cre	Developing T-cells	Commercially available	(72, 73)
Granzyme b-cre	Activated T cells	Commercially available	(74)
Foxp3-cre	T-reg cells	The construct contains a YFP<br>Commercially available	(75)

Vav1-cre	Expressed in all nucleated blood cells Hematopoietic-specific	*Vav*<br>Commercially available	(127)
Agc1-cre ert2	chondrocyte	Cre-dependent body weight and length reduction<br>Commercially available	(79, 80)
Col2-cre	Embryonic chondrocyte	*Col2a1-cre*<br>Commercially available	(81)
Prx1-cre	Limb bud mesenchymal progenitor cell	*Prrx1-cre*<br>Commercially available	(83, 97)
Gli-cre ert2	Periosteum stem cell	Commercially available	(84)
Rank-cre	Osteoclast precursor cells	*Tnfrsf11a*	(85)
muscle CK-cre	Skeletal muscle, cardiomyocyte	Commercially available	(86)
Pax3-cre	muscle cell precursor, and neural, cardiac, etc.	Homozygous is embryonic lethal<br>Commercially available	(87)
Le-cre	cones	*HRGP-cre*; under the control of human *OPN1LW opsin1*<br>*Commercially available*	(88)
MLR10-cre	Lens epithelium and fibres	*Cryaa* (crystallin, alpha A) promoter modified by the insertion of a Pax6 consensus binding element/lens epithelial cell enhancer<br>Commercially available	(88)

Keratin14-cre	Basal layer of (embryonic) epidermis	Human Keratin14 promoter; *K14* or *Krt14*<br>Commercially available	(89)
αMHC-MErcreMer	cardiomyocyte	Commercially available	(93)
Myf5-cre	Dermis; skeletal muscle and brown fat progenitor	Homozygous is perinatal lethal<br>Commercially available	(95)
Ucp1-cre	mature brown adipocytes and white adipocyte upon cold exposure	Commercially available	(96)
Alb-cre	hepatocyte	Commercially available	(92)
Villin-creERT2	Intestinal epithelium	Commercially available	(98)
Villin-cre	Intestinal epithelium	Homozygous mice die prematurely<br>Commercially available	(99)
Six2-cre	Nephron progenitor cell	The strain also expresses *EGFP* and *Six3*<br>Commercially available	(91)
Ksp-cre	Kidney epithelial cells	*Cdh16*<br>Commercially available	(91)
RIP-cre	Pancreas Beta-cell	Commercially available	(100)
PDX-cre ert2	Pancreas Beta-cell		(100)

Fabpl-cre	Distal colon epithelial cells (from E13.5)	Rat *Fabpl*$^{4\times\ at\ -132}$*-Cre*	(142)
Plp-cre ert	Oligodendrocyte; Schwann-cell	Commercially available	(102)
Wnt1-cre	Neural crest	Commercially available	(101)
Sox10-cre	Neural crest		(101)`
Nestin-cre ert2	Neural stem and progenitor cells	Commercially available	(103)
Nestin-cre	Neural stem and progenitor cells	Commercially available	(104, 105)
Nex-cre	Pyramidal cells	*Neurod6*	(106)
CamK2-cre	Postmitotic excitatory forebrain neurons	Commercially available	(107)
AAV-syn-cre	dorsal hippocampus	Commercially available AAV vectors	(110)
AgRP-IRES-Cre	AgRP neurons of the hypothalamus Arcuate nucleus	Commercially available	(112)
Sim1-cre	Sim+ neurons in the PVN of the hypothalamus	Commercially available	(113)
